# Incidental Adrenal Mass in a Patient With Surgically Treated Lung Adenocarcinoma

**DOI:** 10.7759/cureus.19938

**Published:** 2021-11-27

**Authors:** William T Daprano, Seema Shroff, Vladimir Neychev

**Affiliations:** 1 Medicine, University of Central Florida College of Medicine, Orlando, USA; 2 Department of Pathology, AdventHealth Orlando, Orlando, USA; 3 Department of Clinical Sciences, University of Central Florida College of Medicine, Orlando, USA

**Keywords:** adrenal metastasis, adenocarcinoma lung, unilateral adrenalectomy, isolated, laparoscopic adrenalectomy, secondary adrenal cancer, metachronous tumor

## Abstract

Adrenal metastases are not uncommon in patients with widespread metastatic lung cancer. Isolated metachronous adrenal metastases in cases of surgically treated lung cancer without long-term evidence of disease are rare and may pose a diagnostic and treatment dilemma. The current literature suggests that in such cases, adrenalectomy provides better median and overall survival rates. This case presents an incidentally discovered isolated adrenal mass in a patient with a past medical history of lung adenocarcinoma that was surgically removed three years before metastasis discovery. The patient successfully underwent adrenalectomy and was disease-free with no apparent complications at her three-month follow-up visit. The case highlights the importance of long-term radiographic surveillance after surgical resection of lung adenocarcinoma for the prompt diagnosis and timely treatment of metachronous metastases.

## Introduction

Lung cancer is the most common malignancy in the world by incidence in males and second most common malignancy in females [1]. Lung cancer ranks first in worldwide mortality rates in males and the second in females [1]. The high morbidity and mortality rates may be related to the insidious biology of lung cancer. While there is controversy over current screening standards, there is no doubt that most cases of lung cancer are discovered incidentally on unrelated imaging or in symptomatic cases with advanced disease [2,3]. Because of the risks of recurrence or metastasis, it is imperative to continue to monitor lung cancer patients who had curative treatment. Five-year lung cancer survival is 59.8% for localized disease, 32.9% for regional disease spread to lymph nodes, and 6.3% for distant disease metastasis [4]. Common sites of metastases for lung cancer are the nervous system (39%), bone (34%), liver (20%), and adrenal gland (8%) [5]. Isolated metachronous or synchronous adrenal metastasis is rare, with an estimated incidence rate between 1%-6% [6-8]. In the case of isolated adrenal metastasis, metachronous metastasis, lack of lymph node involvement, and disease-free interval are among the factors associated with better survival rates [7,9].

This case presents a patient with an incidentally discovered isolated single right adrenal mass three years after curative surgical resection of right lung adenocarcinoma.

## Case presentation

**Figure 1:**
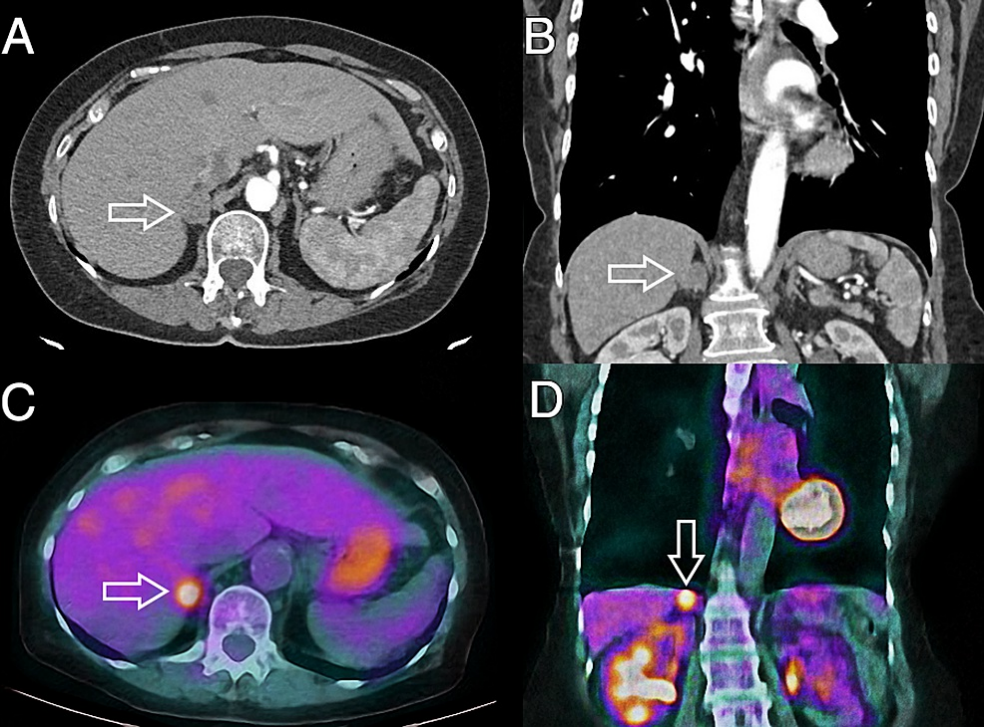
Follow-up diagnostic imaging A) axial and B) coronal computed tomography (CT) images with arrows pointing at the right adrenal tumor. Characteristics of the mass include heterogeneous textures and irregular borders raising radiological suspicion for possible malignancy C) axial and D) coronal positron emission tomography/computed tomography (PET/CT) images with arrows pointing at the right adrenal tumor

A 76-year-old otherwise healthy woman with a history of surgically resected stage 1A right lung adenocarcinoma and no adjuvant chemotherapy had her routine follow-up evaluation with her pulmonologists and oncologist three years postoperatively. Past medical history and social history were unremarkable, with no smoking or alcohol use. Her physical exam was unremarkable with vital signs, laboratory workup, and lung function test within normal limits. The follow-up diagnostic imaging included computerized tomography (CT) (Figure 1A, 1B) and full-body positron emission tomography (PET) (Figure 1C, 1D) that showed an incidental 2 cm hypermetabolic right adrenal mass without any other pathological lesions or concerning regional or distant lymphadenopathy.

A review of a CT scan performed six months prior revealed no right adrenal mass or other suspicious findings. The patient was otherwise healthy with no signs or symptoms concerning a functional adrenal mass or advanced malignancy. She denied abdominal or right flank pain. Biochemical studies for hormonal overproduction, including aldosterone/renin ratio, late-night salivary cortisol, and adrenocorticotropic hormone, and plasma catecholamines with urine metanephrines) were within the physiological ranges. Given the patient’s history, the hypermetabolic activity of the mass, and the likelihood of rapid growth of the mass to 2.5 cm within six months, metastatic disease was high on the differential. Other options considered due to the fast growth rate included adrenocortical carcinoma and less likely adrenal adenoma. Because this was an isolated, quickly growing adrenal mass with no lymphadenopathy, a decision was made to proceed with the right adrenalectomy as the treatment of choice for a definitive resection and histological diagnosis. The patient underwent successful laparoscopic right adrenalectomy.

Procedure in brief

**Figure 2:**
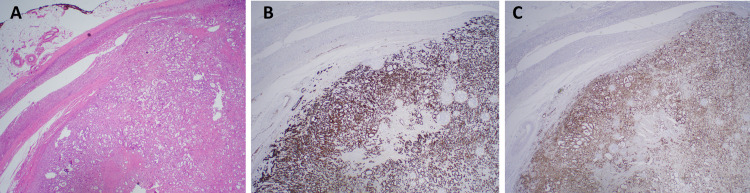
Histology and immunohistochemistry of the right adrenal gland with the tumor A) Representative hematoxylin and eosin (H&E) stain of the tumor showing normal adrenal gland top left and tumor center and right side; B) and C) Immunohistochemistry (IHC) images of tumor cells positive for cytokeratin 7 (CK7) and Napsin A, respectively consistent with pulmonary origin. The adrenal tissue is negative for both these markers.

After the initiation of general endotracheal anesthesia, the patient was placed in the right decubitus position, and operative fields prepped and draped in a standard sterile fashion. A right subcostal approach with four trocars was used. A harmonic scalpel was used to detach the right triangular ligament of the liver, enabling medial rotation and retraction of the right hepatic lobe with a laparoscopic fan retractor. Using hook electrocautery and a harmonic scalpel the retroperitoneum was opened and dissection was carried upwards toward the diaphragm. The lateral border of the inferior vena cava was defined after meticulous dissection around the medial aspect of the right adrenal gland and tumor. After careful dissection and clipping of the right adrenal central vein, the vein was transected close to the adrenal gland with harmonic scalpel. The adrenal gland with the tumor was then mobilized inferiorly, laterally, superiorly, and finally posteriorly until the entire gland with the tumor and a rim of surrounding fatty tissue were completely mobilized. The gland was removed from the peritoneal cavity with an EndoCatch bag and sent to pathology as a permanent specimen. There was minimal blood loss throughout the case, and the final inspection of the operative field reviled no bleeding or other issues. The patient tolerated the procedure well without complications. The final surgical pathology report revealed a 3.5 x 2.2 x 1.5 cm adrenal gland that demonstrated a 2.5 x 2.2 x 1.8 cm variegated ivory-tan to yellow, firm, well-delineated mass involving the adrenal cortex and medulla. Immunohistochemical staining of cells from the mass was positive for cytokeratin 7 (CK7), TTF1, and Napsin-A (Figure 2). Immunohistochemistry (IHC) for cytokeratin 20 (CK20) was negative. These results and the patient’s prior medical history were consistent with metastatic lung adenocarcinoma. Postoperatively, the patient recovered as expected and required no additional treatments at this time. At two-week and three months follow-up, she reported no issues or concerns.

## Discussion

The main sites for lung cancer metastasis include the brain and bony skeleton. The adrenal gland is one of the less common sites for metastasis. Patients with metastasis secondary to lung adenocarcinoma more commonly present with widespread synchronous metastatic disease in the lungs, bone, brain, and liver [4,5]. On the other hand, isolated incidentally found adrenal masses in a patient with a history of malignancy are not always metastatic disease; in fact, adrenal adenomas are more common than metastatic disease [4,9]. Thus, cases with previously treated lung cancer and incidentally found isolated adrenal mass can pose a significant diagnostic dilemma and management challenge. Fine needle aspiration (FNA) of these masses is not recommended due to limited diagnostic and therapeutic potential and possible complications of such biopsy [10,11]. The initial workup of any adrenal incidentaloma includes biochemical testing to assess for steroid or catecholamine hormonal overproduction [12]. The choice of surgical versus nonsurgical treatment approaches depends on many factors, including past medical, surgical, and social histories, as well as adrenal tumor biology, anatomy, radiological features, and growth rate. In the past, nonsurgical management of adrenal metastases has demonstrated a significantly decreased survival benefit when compared to adrenalectomy. It has been shown that surgically managed patients with isolated adrenal metastasis from primary non-small cell lung cancer had a five-year survival rate of 34% while the five-year survival rate for non-surgically managed patients was 0% [2]. The advantages that laparoscopic adrenalectomy provides over an open approach include the following: improved recovery time, decreased morbidity, better cosmesis, and patient satisfaction [13]. Thus, after functional assessment of the mass, the physician should recommend laparoscopic adrenalectomy for the treatment of all isolated incidental adrenal masses discovered on imaging when possible [8,14]. A greater than six-month disease-free interval (DFI) from initial cancer diagnosis is widely accepted as an important prognostic factor when determining survival [15,16].

Our patient presented with an isolated rapidly, growing, functionally inactive 2 cm adrenal mass without any other sites of synchronous metastatic disease that made her a candidate for curative right adrenalectomy. Postoperative diagnostic follow-up and monitoring with whole-body CT and positron emission tomography (PET)/CT scans should continue annually because the prompt detection and curative resection of incidental metastasis found after primary lung adenocarcinoma resection provides a significant survival benefit [2]. This case adds to the accumulating evidence that proper monitoring for malignancy recurrence and laparoscopic resection of suspected adrenal metastasis offers survival benefits. Long-term annual follow-up imaging and monitoring using high-resolution CT for four years post-surgical resection and low-dose CT after four years of radiographic surveillance is recommended for prompt diagnosis and timely treatment [17,18].

## Conclusions

Isolated adrenal metastasis may occur in lung cancer patients' years after curative resection of the primary tumor. Long-term annual follow-up imaging and monitoring are recommended for prompt diagnosis and timely treatment. When possible, patients with isolated metachronous metastatic disease should be offered a curative resection that confers a significant survival benefit.
